# Inducing Melanoma Cell Apoptosis by ERp57/PDIA3 Antibody in the Presence of CPI-613 and Hydroxychloroquine

**DOI:** 10.7150/jca.92252

**Published:** 2024-02-04

**Authors:** Naohisa Ichiki, Chiemi Saigo, Yuki Hanamatsu, Hiroaki Iwata, Tamotsu Takeuchi

**Affiliations:** 1 Department of Dermatology, Gifu University Graduate School of Medicine, Gifu, Japan.; 2 Department of Pathology and Translational Research, Gifu University Graduate School of Medicine, Gifu, Japan.; 3 The United Graduate School of Drug Discovery and Medical Information Sciences, Gifu University, Gifu, Japan.; 4 Center for One Medicine Innovative Translational Research; COMIT, Gifu University, Gifu, Japan.

**Keywords:** melanoma, CPI-613, ERp57/PDIA3, hydroxychloroquine, monoclonal antibody

## Abstract

The combination of the cancer mitochondrial metabolic inhibitor CPI-613 and hydroxychloroquine has tumor-suppressive effects on clear cell sarcoma, which shares pathobiological properties with melanoma. Therefore, we intended to examine the effects of a combination of CPI-613 and hydroxychloroquine on the growth of melanoma cells in the present study. However, cell death was not induced in melanoma cells. Therefore, a monoclonal antibody, ICT, that induced apoptosis in melanoma cells in combination with CPI-613 and hydroxychloroquine was developed. Immunoprecipitation, mass spectrometry, and small interfering RNA (siRNA)-mediated gene silencing demonstrated that ICT targeted Endoplasmic Reticulum Resident Protein 57/ Protein Disulfide Isomerase Family A Member 3 (ERp57/PDIA3), which was first identified as being upregulated by metabolic depletion stress and is localized on the cell surface during immunogenic cell death. The combination of CPI-613 and hydroxychloroquine enhanced the localization of ERp57/PDIA3 to the surface of melanoma cells. siRNA-mediated downregulation of ERp57/PDIA3 did not significantly induce ICT-mediated apoptosis in melanoma cells in the presence of CPI-613 and hydroxychloroquine. Therefore, the ICT antibody acts as a tumor suppressor in melanoma cells by targeting the cell membrane ERp57/PDIA3, expression of which was enhanced by the combination of CPI-613 and hydroxychloroquine.

## Introduction

Cutaneous melanoma is a common cancer, and its incidence has steadily increased since the mid-1970s 1. Despite recent advances, a large proportion of patients with melanoma do not benefit from existing first-line combination immunotherapies and targeted therapies 2-4. In addition, dual immune checkpoint inhibition with ipilimumab plus nivolumab has shown clinical efficacy and is a highly toxic treatment for advanced melanoma 5. Therefore, further development of therapeutic options for patients with melanoma is required.

Most malignant cells, including melanoma cells, show enhanced metabolic activity through the translocation of metabolism-related molecules, such as glucose transporter 1 (GLUT-1) from intracellular membranes to the cell surface 6. Targeting these metabolic molecules is an attractive therapeutic approach in the era of precision oncology 7, 8. Devimistat (CPI-613) is a lipoic acid antagonist that inhibits mitochondrial energy metabolism and induces apoptosis in various cancer cells 9-12. The inhibitory effects of a combination of CPI-613 and hydroxychloroquine (CPI-613/HCQ) on the progression of clear cell sarcoma (CCS) have been reported 13, and a clinical trial investigating the effects of CPI-613/HCQ was conducted in patients with relapsed or refractory clear cell sarcoma [Clinical Trials. gov ID: NCT04593758; https://clinicaltrials.gov/study/NCT04593758].

CCS is also termed “melanoma of the soft part” 14 based on the pathobiological and histopathological immunoprofiling similarities between CCS and cutaneous melanoma 15, 16. This study determined if the inhibitory effects of CPI-613/HCQ observed on CCS cells are also observed on melanoma cells and subsequently explored a method to induce apoptosis in melanoma cells in the presence of CPI-613/HCQ.

## Materials and methods

### Cells and culture

A2058, G361, and Mewo melanoma cell lines were obtained from the Japan Health Science Research Resources Bank (Osaka, Japan). The cells were cultured in Dulbecco's Modified Eagle's Medium (Gibco BRL, Life Technologies, Grand Island, NY, USA) containing 10% heat-inactivated fetal bovine serum. The cells were passaged for no more than 6 months after resuscitation.

### CPI-613, hydroxychloroquine, and double staining with annexin V and propidium iodide

CPI-613 and hydroxychloroquine were purchased from AdooQ BioScience (Irvine, CA, USA) and FUJIFILM Wako Pure Chemical Corporation (Osaka, Japan), respectively.

The number of cells undergoing apoptosis was quantified using FITC-conjugated annexin V and propidium iodide (PI; PromoCell GmbH, Heidelberg, Germany). Briefly, 5 × 10^4^ cells were harvested under different culture conditions, washed, resuspended in binding buffer, mixed with annexin V-FITC and PI, and analyzed as previously described 17. In several experiments, we examined the apoptotic status of non-treated cells.

### Generation of monoclonal antibodies

The experimental protocol was approved by the Animal Care Committee of the Gifu University Graduate School of Medicine (approval no. 2021-149, Gifu, Japan). Briefly, BALB/c mice were intraperitoneally immunized weekly with 1 × 10^7^ Mewo cells. Monoclonal antibodies were generated according to the modified Köhler and Milstein method, as previously described 18, 19. Hybridoma clones were screened using a two-step process, as indicated below, and cloned via limiting dilution. Clones that produced an antibody reacting with the surface membrane of Mewo cells were selected using immunofluorescent staining, as described below. The candidate antibodies that induced apoptosis in the presence of CPI-613/HCQ were identified. The antibody subclasses were determined using an isotyping kit (Pharmaceuticals, Canton, MA, USA). The antibody was purified from the culture supernatant using Immuno-Assist MG-PP (Kanto Chemical, Tokyo, Japan).

### Immunofluorescence staining

Immunofluorescence staining was performed as previously described 12. Briefly, the cells were incubated with the antibodies for 30 min at 4 °C. After washing, the cells were incubated with Alexa Fluor 488 goat anti-mouse IgG (H+L) (A11001; Invitrogen, Carlsbad, CA, USA) for 30 min at 4 °C. The stained cells were analyzed using a Guava EasyCyte cell analyzer (Hayward, CA, USA).

### Immunoprecipitation, peptide mass fingerprinting, and mass spectrometry

Immunoprecipitation, peptide mass fingerprinting, and mass spectrometry were conducted as previously described 20. Briefly, the purified ICT antibody was bound to M‐270 epoxy magnetic beads (Dynabeads® Antibody Coupling Kit, Life Technologies, Carlsbad, CA) according to the manufacturer's protocol. The ICT‐bound protein band from Mewo or A2058 cell lysates was digested with trypsin and subjected to matrix‐assisted laser desorption ionization time‐of‐flight analysis (Microflex LRF 20; Bruker Daltonics, Germany). Spectra were collected at 300 shots per spectrum over a range of 700-4,000 m/z and calibrated using two-point internal calibration and trypsin autodigestion peaks (842.5099 m/z and 2,211.1046 m/z). The peak list was generated using Flex Analysis software (version 3.0; Bruker Daltonics). The thresholds used for identifying appropriate peaks were 500 for the minimum resolution of monoisotopic mass and 6 for S/N. The search program MASCOT, developed by Matrix Science (http://www.matrixscience.com/), was used for protein identification via peptide mass fingerprinting. The following parameters were used for the database search: trypsin as the cleaving enzyme, a maximum of one missed cleavage, iodoacetamide (Cys) as complete modification, oxidation (Met) as partial modification, monoisotopic masses, and a mass tolerance of ±0.2 Da.

### Small interfering RNA‐mediated RNA interference

The detailed procedure for small interfering RNA (siRNA) silencing of target genes has been described previously 20. siRNAs (4392420, Assay s6228 and s6229, Thermo Fisher Scientific, Rockford, IL, USA) were used to silence the* ERp57/PDIA3* gene, while a Trilencer-27 Universal scrambled negative control siRNA-duplex (OriGene, Rockville, MD, USA) was used as a non-silencing control. The siRNAs were transfected into cells using Lipofectamine RNAiMAX (Invitrogen), according to the manufacturer's instructions.

### Quantitative real-time reverse transcription polymerase chain reaction

cDNA was synthesized from total RNA, and polymerase chain reaction (PCR) was performed using a reverse transcription PCR (RT-PCR) kit (Takara, Seta, Japan) 12. The procedure was performed according to the manufacturer's instructions. Real-time PCR was performed using the SYBR Green reaction kit (Roche Diagnostics, GmbH, Mannheim, Germany) in a LightCycler (Roche Diagnostics) according to the manufacturer's instructions. The following primers were used for real-time RT-PCR:

ERp57/PDIA3-forward 5′-TTGATTGCACTGCCAACACT-3′;

ERp57/PDIA3-reverse 5′-AGTTGCTGGCTGCTTTTAGG-3′;

GAPDH-forward 5′-GAAGGTGAAGGTCGGAGTC-3′;

GAPDH-reverse 5′-GAAGATGGTGATGGGATTTC-3′.

The expression of each target gene was analyzed using the 2^-ΔΔCT^ method described by Livak and Schmittgen 21 using the LightCycler system. The ΔCT values were normalized to GAPDH in both the Trilencer-27 Universal scrambled negative control siRNA (control) and si-ERp57/PDIA3-treated groups. The values for the si-ERp57/PDIA3-treated group were calculated for each target gene as the fold change relative to the control group (set to 1.0).

### Immunoblotting

Immunoblotting was performed according to the method described by Towbin et al., with modifications as previously described 22, 23. The cell lysates were electrophoresed on sodium dodecyl sulfate-polyacrylamide gels and electroblotted onto polyvinylidene difluoride membranes (Immobilon-FL transfer membranes; Millipore, Bedford, MA, USA). The membrane was blocked with Block Ace (blocking milk; Yukijirushi, Sapporo, Japan) and subsequently incubated with 1.0 μg/mL ICT and rabbit monoclonal anti-sodium potassium ATPase antibody (EP1845Y; Abcam, Cambridge, UK). Goat anti-rabbit IgG highly cross-adsorbed secondary antibody Alexa Fluor Plus 800 (A32735, Invitrogen; Thermo Fisher Scientific, Inc., Waltham, MA, USA) and goat anti-Mouse IgM (heavy chain) secondary antibody Alexa Fluor 647 (A-21238, Invitrogen; Thermo Fisher Scientific, Inc.) were used for fluorescence immunodetection.

Fluorescent signals were detected using the Invitrogen iBright 1500 gel imaging system (Thermo Fisher Scientific).

## Results

### Generation of ICT

**Figure 1:**
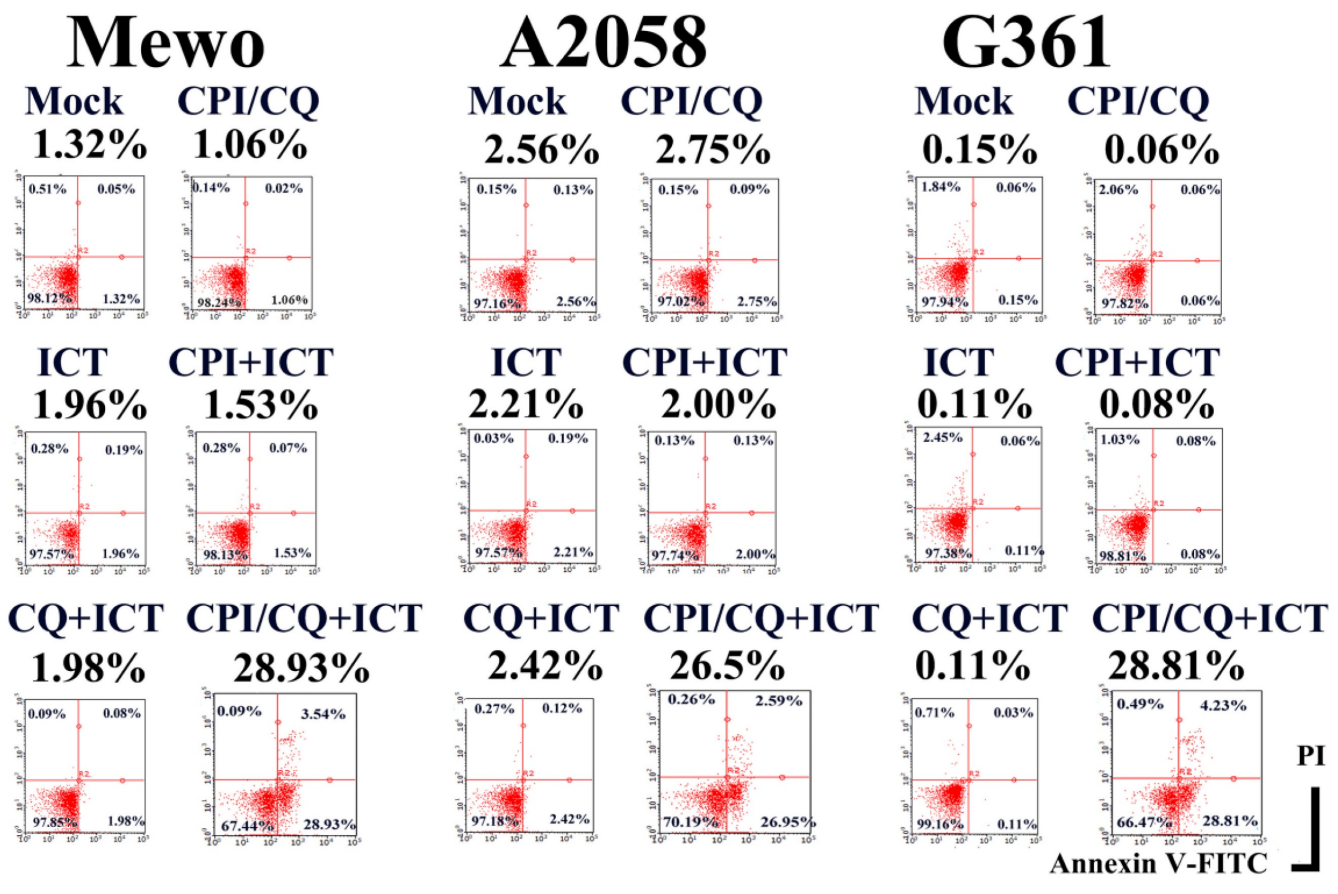
**ICT with CPI-613 and hydroxychloroquine induces apoptosis in melanoma cells.** ICT antibody-induced apoptosis in Mewo, A2058, and G361 melanoma cells in the presence of CPI/HCQ. In contrast, CPI/HCQ without ICT, ICT alone, ICT with CPI, and ICT with HCQ did not induce significant apoptosis in melanoma cells. Cells were treated with 1 μg/mL CPI-613, 10 μg/mL hydroxychloroquine, and/or 1 μg/mL of ICT antibody for 16 h. Double staining with fluorescence-conjugated annexin V and propidium iodide was performed using a cell analyzer. Percent of Annexin V-positive PI-negative fraction is indicated under the treatment title, for example 28.93% in CPI-613/HCQ+ICT treated Mewo cells.

CPI-613/HCQ induced apoptosis in <3% of melanoma cells. We successfully generated a monoclonal antibody designated ICT (ICT was a serial hybridoma clone number in our laboratory) that induced apoptosis of melanoma cells in the presence of CPI-613/HCQ, whereas ICT alone did not induce apoptosis in melanoma cells (Figure 1).

### ICT recognized the ERp57/PDIA3

**Figure 2:**
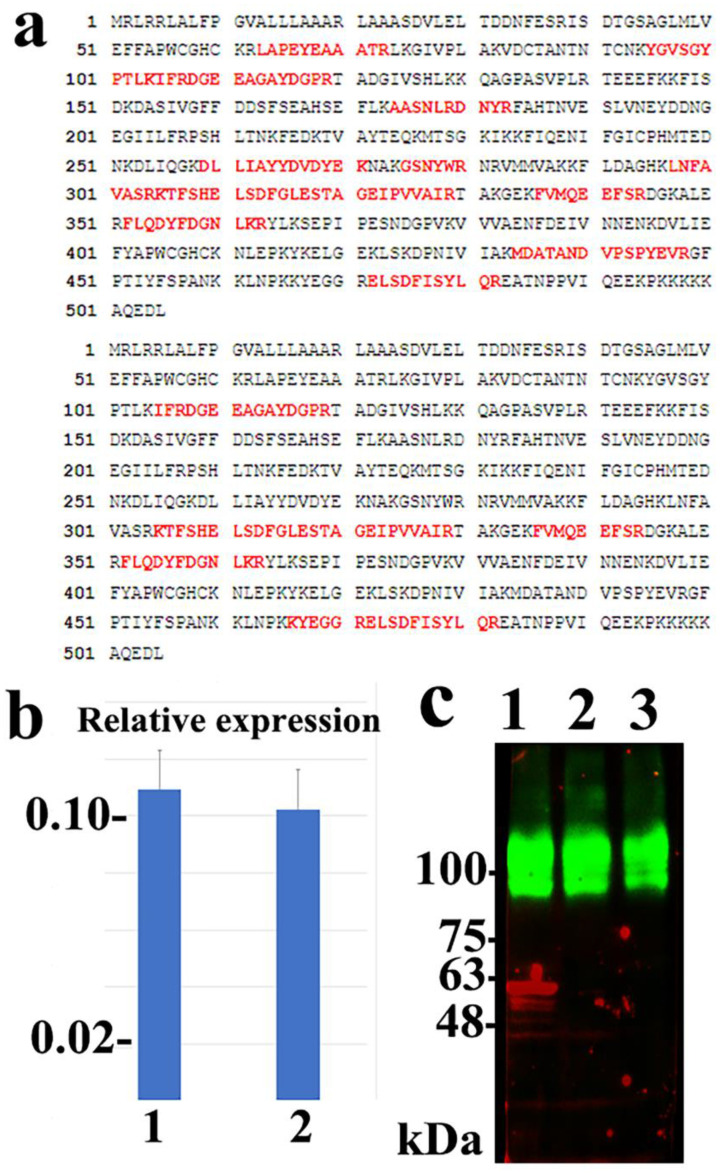
** Identification of ERp57/PDIA3 as the protein recognized by ICT.** (a) Peptide coverage is present in human ERp57/PDIA3 from the ICT antigen isolated from Mewo (upper) and A2058 (lower) cells. The matched peptides are shown in red. (b) Quantitative RT-PCR reveals that siRNA-mediated silencing using the s6228 (lane 1) or s6229 (lane 2) siRNA-duplex successfully reduced the mRNA expression of *ERp57/PDIA3* in Mewo melanoma cells compared to that in control cells (Trilencer-27 Universal scrambled negative control siRNA duplex) (control set to 1.0). (c) Fluorescent immunoblotting shows that siRNA-mediated silencing of ERp57/PDIA3 resulted in the reduction of the red ICT protein band (lane 1, Trilencer-27 Universal scrambled negative control siRNA-duplex; lane 2, s6228 siRNA; lane 3, s6229 siRNA) in Mewo cells. The green band represents the sodium potassium ATPase protein.

The ICT antigen was purified from the Mewo and A2058 cells using ICT-binding M-270 epoxy magnetic beads. A protein band of approximately 60 kDa was detected via immunoblotting and Coomassie brilliant blue staining using ICT-binding M-270 epoxy magnetic beads, but not when the control murine IgM-binding M-270 epoxy magnetic beads were used. The 60-kDa band was cut and analyzed using peptide mass fingerprinting and appeared to be human ERp57/PDIA3 (Figure 2a). The experiment was repeated using A2058 cells and consistent results were obtained (Supplementary Figure 1). siRNA silencing of the *ERp57/PDIA3* gene led to a decreased 60-kDa ICT band on immunoblotting in Mewo cells (Figure 2c). These results suggest that ICT recognizes the ERp57/PDIA3 protein.

### Functions of ERp57/PDIA3 in CPI-613/HCQ-treated melanoma cells

**Figure 3:**
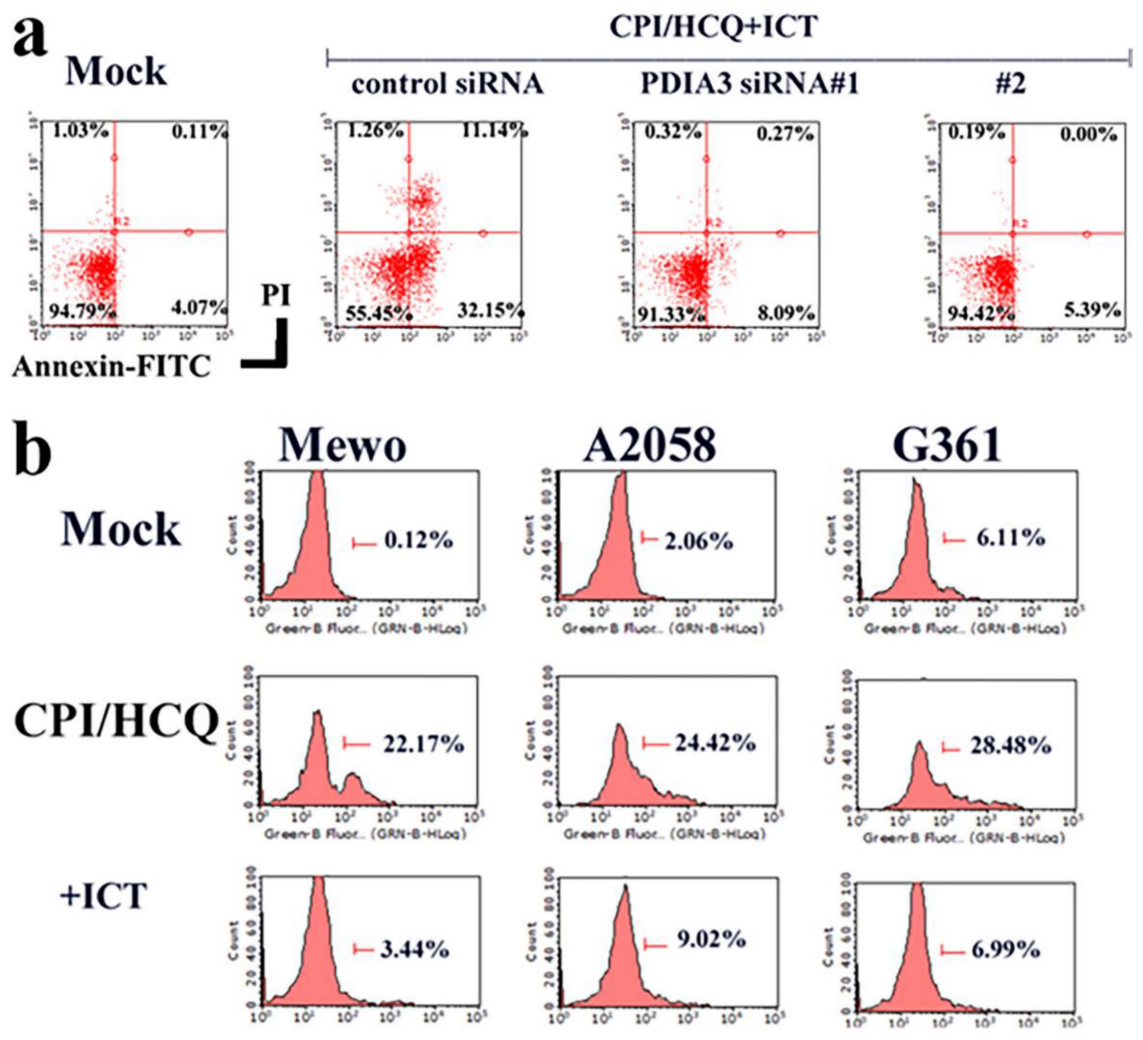
** Function of ERp57/PDIA3 in CPI-613/HCQ-treated melanoma cells.** (a) The downregulation of ERp57/PDIA3 blocked ICT-induced apoptosis in Mewo melanoma cells. (b) CPI-613/HCQ induces the cell surface localization of ERp57/PDIA3 in melanoma cells, and ICT eliminates the cell surface expression of ERp57/PDIA3. The result without CPI-613/HCQ or ICT antibody treatment is shown in Mock.

The downregulation of Erp57/PDIA3 inhibited ICT-induced apoptosis in Mewo melanoma cells (Figure 3a). CPI-613/HCQ induced the cell surface localization of Erp57/PDIA3 in melanoma cells, and ICT eliminated the cell surface expression of Erp57/PDIA3 (Figure 3b).

## Discussion

CPI-613 is an alpha-lipoic acid analogue that targets the mitochondrial enzymes pyruvate dehydrogenase and alpha-ketoglutarate dehydrogenase 9. Multiple clinical trials regarding CPI-613 are currently underway, and it has been granted orphan drug designation by the U.S. Food and Drug Administration for the treatment of several malignant tumors, including soft tissue sarcomas such as CCS 9-11. In combination with chemotherapy or hydroxychloroquine, CPI-613 has been shown to reduce tumor progression 7, 13.

The inhibitory effects of CPI-613/HCQ on CCS have been reported previously 13. In this study, it was hypothesized that CPI-613/HCQ could induce cell death in melanoma cells, but sufficient cell death was not observed in CPI-613/HCQ-treated melanoma cells. Although CCS has pathobiological similarities with melanoma, the *EWS/ATF1* fusion gene, which characterizes most CCS, may lead to distinct molecular differences 16.

Recent advances have shown that several malignant tumors, including melanoma, enhance their metabolic activity via the translocation of metabolism-related molecules, such as GLUT1, from intracellular membranes to the cell surface 8. Therefore, it was hypothesized that targeting the surface membrane molecules induced by CPI-613/HCQ may induce cell death in melanoma cells.

In this study, ICT, an anti-ERp57/PDIA3 antibody, induces apoptosis in melanoma cells treated with CPI-613/HCQ. ERp57/PDIA3 is a pleiotropic member of the protein disulfide isomerase (PDI) family that is predominantly located in the endoplasmic reticulum, though it has been located in other cellular compartments, such as the cell surface membrane 24, 25. ERp57/PDIA3, also known as GRP58, increases after glucose depletion 26. Notably, PDI inhibitors induce apoptosis in several types of cancer 27. CPI-613/HCQ treatment leads to metabolic depletion, followed by the relocation or accumulation of ERp57/PDIA3 on the cell surface membrane in melanoma cells. ICT antibodies may target accumulated cell surface membrane ERp57/PDIA3 or ERp57/PDIA3 bound to cell surface membrane proteins. We speculate that the localization of ERp57/PDIA3, or other cell surface membrane molecules, the expression of which is chaperoned by ERp57/PDIA3, might be important for rescuing melanoma cells from metabolic depletion-induced apoptosis. Alternatively, ICT antibodies could exert direct cell-toxic effects by targeting cell surface ERp57/PDIA3 and/or ERp57/PDIA3-bound molecules. A subsequent study will be conducted to elucidate the mechanism by which ICT induces apoptosis in melanoma cells in the presence of CPI-613/HCQ. Therefore, ICT antibodies in combination with CPI-613/HCQ could be applicable for treating various malignant tumors other than melanoma. This includes not only those tumors in which ERp57/PDIA3 is putatively expressed on the cell surface membrane (i.e., T-cell leukemia 28 and breast cancer 29), but also in tumors expressing no significant cell surface ERp57/PDIA3. Experiments to investigate the therapeutic effect of the combination of the ICT antibody and CPI-613/HCQ in mouse melanoma models are ongoing.

Notably, the present study also demonstrated that the ICT antibody induced apoptosis in BRAF wild-type Mewo melanoma cells as well as in BRAF-mutated A2058 and G361 cells in the presence of CPI-613/HCQ. Therefore, the combination of an ICT antibody and CPI-613/HCQ may be useful for patients with BRAF wild-type melanoma who do not benefit from BRAF and MEK inhibitors. However, further studies, including clinical trials, are required to verify this hypothesis.

In conclusion, this study revealed that ICT, an ERp57/PDIA3 antibody, induces apoptosis in melanoma cells treated with CPI-613/HCQ.

## Supplementary Material

Supplementary figure.

## Funding

This study was supported by grants from the Japanese Ministry of Education (KAKEN 23K06423).
