# Supplementary material for: Inducing Melanoma Cell Apoptosis by ERp57/PDIA3 Antibody in the Presence of CPI-613 and Hydroxychloroquine
Source: J Cancer. 2024 Feb 4;15(7):1779–85. doi: 10.7150/jca.92252 (PMC10905412; doi:10.7150/jca.92252)
Supplement: Supplementary file 1 — Supplementary figure. [file jcav15p1779s1.pdf]

1	MRLRRLALFP	GVALLLAAAR	LAAASDVLEL	TDDNFESRIS	DTGSAGLMLV
51	EFFAPWCGHC	KRLAPEYEAA	ATRLKGIVPL	AKVDCTANTN	TCNKYGVSGY
101	PTLKIFRDGE	EAGAYDGPRT	ADGIVSHLKK	QAGPASVPLR	TEEEFKKFIS
151	DKDASIVGFF	DDSFSEAHSE	FLKAASNLRD	NYRFAHTNVE	SLVNEYDDNG
201	EGIILFRPSH	LTNKFEDKTV	AYTEQKMTSG	KIKKFIQENI	FGICPHMTED
251	NKDLIQGKDL	LIAYYDVDYE	KNAKGSNYWR	NRVMMVAKKF	LDAGHKLNFA
301	VASRKTFSHE	LSDFGLESTA	GEIPVVAIRT	AKGEKFVMQE	EFSRDGKALE
351	RFLQDYFDGN	LKRYLKSEPI	PESNDGPVKV	VVAENFDEIV	NNENKDVLIE
401	FYAPWCGHCK	NLEPKYKELG	EKLSKDPNIV	IAKMDATAND	VPSPYEVRGF
451	PTIYFSPANK	KLNPKKYEGG	RELSDFISYL	QREATNPPVI	QEEKPKKKKK
501	AQEDL				

Supplementary Figure 1

The ICT antigen was also purified from the A2058 cells and analyzed using peptide mass fingerprinting. It again appeared to be human ERp57/PDIA3. The matched peptides are shown in red.
